# Antimicrobial Disk Susceptibility Testing of *Leptospira* spp. Using *Leptospira* Vanaporn Wuthiekanun (LVW) Agar

**DOI:** 10.4269/ajtmh.15-0180

**Published:** 2015-08-05

**Authors:** Vanaporn Wuthiekanun, Premjit Amornchai, Sayan Langla, Nicholas J. White, Nicholas P. J. Day, Direk Limmathurotsakul, Sharon J. Peacock

**Affiliations:** Mahidol-Oxford Tropical Medicine Research Unit, Faculty of Tropical Medicine, Mahidol University, Bangkok, Thailand; Nuffield Department of Medicine, Centre for Tropical Medicine and Global Health, University of Oxford, Headington, Oxford, United Kingdom; Department of Tropical Hygiene, Faculty of Tropical Medicine, Mahidol University, Bangkok, Thailand; Department of Medicine, University of Cambridge, Addenbrooke's Hospital, Cambridge, United Kingdom

## Abstract

*Leptospira* Vanaporn Wuthiekanun (LVW) agar was used to develop a disk diffusion assay for *Leptospira* spp. Ten pathogenic *Leptospira* isolates were tested, all of which were susceptible to 17 antimicrobial agents (amoxicillin/clavulanic acid, amoxicillin, azithromycin, cefoxitin, ceftazidime, ceftriaxone, chloramphenicol, ciprofloxacin, clindamycin, doripenem, doxycycline, gentamicin, linezolid, nitrofurantoin, penicillin, piperacillin/tazobactam, and tetracycline). All 10 isolates had no zone of growth inhibition for four antimicrobials (fosfomycin, nalidixic acid, rifampicin, and trimethoprim/sulfamethoxazole). Of the ten *Leptospira*, seven had a growth inhibition zone of ≤ 21 mm for aztreonam, the zone diameter susceptibility break point for Enterobacteriaceae. This assay could find utility as a simple screening method during the epidemiological surveillance of antimicrobial resistance in *Leptospira* spp.

Leptospirosis is a worldwide zoonotic infection caused by pathogenic members of the genus *Leptospira*. Clinical manifestations range from a mild influenza-like illness to multiorgan failure and death, although the most common clinical presentation is an undifferentiated febrile illness.^1^,^2^ Antibiotics should be commenced as soon as leptospirosis is suspected, using high-dose intravenous penicillin for patients with severe leptospirosis and oral agents such as doxycycline or amoxicillin for milder cases.^3^ Third generation cephalosporins such as ceftriaxone and cefotaxime, and fluoroquinolone antibiotics may also be effective.^3^

There is currently no accepted standard method for assessing the in vitro activity of antimicrobial agents against *Leptospira* species. Routine laboratories do not culture leptospires because of their very slow growth rate and need for specialist expertise, but the recent development of a solid culture medium (*Leptospira* Vanaporn Wuthiekanun [LVW] agar) led to the description of susceptibility testing of *Leptospira* spp. using the Etest method.^4^ Here, we report the results of a pilot study in which we used LVW agar to determine the feasibility of the disk diffusion method for pathogenic *Leptospira*.

Ten *Leptospira* isolates representing four species were tested: seven *Leptospira interrogans* (three serovar Autumnalis, and one each of serovars Bataviae, Canicola, Medanensis, and Pyrogenes), and one *L. borgpetersenii* (Javanica), *L. kirschneri* (Grippotyphosa), and *L. weilii* (Mengdeng). All organisms were maintained in LVW agar tubes at room temperature, as described previously.^5^ One milliliter EMJH broth with 3% rabbit serum was added into the tube, left in air at 30°C for 1 week, and the surface fluid then transferred into 12 mL EMJH broth and incubated at 30°C to reach a final concentration at 10^8^ CFU/mL (assessed by dilution colony counts on solid agar). LVW agar was prepared as previously described,^4^ and contained 1% Noble agar base (Becton Dickinson), 10 mg/L sodium pyruvate (Merck), 2.3 g/L *Leptospira* Medium Base EMJH (Difco), 100 mL/L *Leptospira* Enrichment EMJH (Difco), and 10% rabbit serum (Gibco). Twenty-five mL of LVW agar was poured into a 90-mm diameter petri dish to a depth of 4 mm.

The antimicrobial agents selected for testing (*N* = 22) represent the spectrum of drugs used in tropical settings for the treatment of suspected bacterial sepsis. Disk susceptibility testing was performed by spread plating 300 μL of each isolate (10^8^ CFU/mL) across the surface of a LVW agar plate. These were preincubated at 30°C in 5% CO_2_ for 2 days (the established optimal incubation conditions for LVW agar), after which a standard disk was applied in the center of a single plate for the following antimicrobials (disk content): amoxicillin/clavulanic acid (20/10 μg), amoxicillin (10 μg), azithromycin (15 μg), aztreonam (30 μg), cefoxitin (30 μg), ceftazidime (30 μg), ceftriaxone (30 μg), chloramphenicol (30 μg), ciprofloxacin (5 μg), clindamycin (2 μg), doripenem (10 μg), doxycycline (30 μg), fosfomycin (50 μg), gentamicin (10 μg), linezolid (30 μg), nalidixic acid (30 μg), nitrofurantoin (300 μg), penicillin (10 units), piperacillin/tazobactam (100/10 μg), rifampicin (5 μg), tetracycline (30 μg), and trimethoprim/sulfamethoxazole (1.25/23.75 μg) (Oxoid Ltd, Basingstoke, United Kingdom). An additional plate (without discs) was used as a growth control. Plates were then incubated at 30°C in air and observed every day for 7 days. The growth inhibition zone sizes were measured at the point at which a bacterial lawn was clearly discernible by the naked eye (usually at day 7) (Figure 1). As disk diffusion testing has not been performed previously for *Leptospira*, we used Clinical and Laboratory Standards Institute (CLSI) performance standards (M100-S25) for threshold zone sizes primarily for Enterobacteriaceae, extending to *Pseudomonas aeruginosa* or *Staphylococcus* spp. where zone sizes were not available for the drug being tested (Supplemental Table 1). The results for four antimicrobials (penicillin, doxycycline, ceftriaxone, and chloramphenicol) were also compared with susceptibility testing using a published minimum inhibitory concentration (MIC) method (Etest),^4^ which was performed in parallel with disk testing.

**Figure 1.**
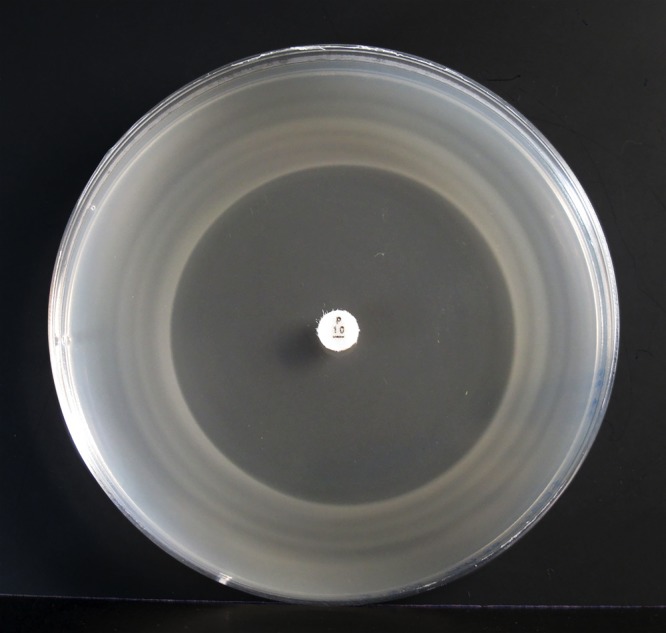
Zone of inhibition (50 mm) for penicillin G disk diffusion method on *Leptospira* Vanaporn Wuthiekanun (LVW) agar for *Leptospira interrogans* serovar Autumnalis strain NR-20161. The plate was prepared by spreading 300 μL of 10^8^ CFU/mL and preincubating at 30°C in 5% CO_2_ for 2 days followed by application of the disk and further incubation at 30°C in air for a total of 7 days.

All 10 *Leptospira* isolates were susceptible to 17 antimicrobials (amoxicillin/clavulanic acid, amoxicillin, azithromycin, cefoxitin, ceftazidime, ceftriaxone, chloramphenicol, ciprofloxacin, clindamycin, doripenem, doxycycline, gentamicin, linezolid, nitrofurantoin, penicillin, piperacillin/tazobactam, and tetracycline) (Table 1). All 10 isolates had no zone of growth inhibition for four antimicrobials (fosfomycin, nalidixic acid, rifampicin, and trimethoprim/sulfamethoxazole) (Table 1). Of the 10 *Leptospira*, seven had a growth inhibition zone of ≤ 21 mm for aztreonam, the zone diameter susceptibility break point of Enterobacteriaceae. Comparison between disk and Etest results for penicillin, doxycycline, ceftriaxone, and chloramphenicol showed concordance between the two methods (all susceptible).

Since LVW agar was developed, it has found use for the isolation of *Leptospira* from the environment,^6^ for long-term maintenance of the organism in agar tubes (> 1 year) without frequent media transfer,^5^ and for susceptibility testing using the Etest method.^4^ In this preliminary evaluation, the disk diffusion method was performed with an individual single antimicrobial disk per LVW agar plate, since preliminary testing demonstrated very large zones of inhibition. Break points have not been established for *Leptospira*, but four antimicrobial agents were apparently inactive and gave no inhibition zones. These drugs may prove useful as inhibitors of contamination in clinical and environmental samples, and could be incorporated in selective *Leptospira* culture media. The findings of our study are consistent with prior reports (using broth MIC) of *Leptospira* susceptibility to amoxicillin, azithromycin, cefoxitin, ceftriaxone, chloramphenicol, ciprofloxacin, doxycycline, erythromycin, and tetracycline^7^ and resistance to fosfomycin, trimethoprim, and sulfamethoxazole.^8^ The disk diffusion method is easy to perform and could become a useful, initial screening test for the epidemiological surveillance of antimicrobial resistance.

## Supplementary Material

Supplemental Table.

## Figures and Tables

**Table 1:** Zone diameter (millimeters) of the 10 *Leptospira* isolates tested

Species	Serovars	Strains	Amoxicillin/clavulanic acid†	Amoxicillin†	Aztreonam†	Cefoxitin†	Ceftazidime†	Ceftriaxone†
*Leptospira interrogans*	Autumnalis	L0013	85	85	57	85	85	77
*L. interrogans*	Autumnalis	L0752	85	85	30	85	85	70
*L. interrogans*	Autumnalis	NR-20161*	77	73	16	70	67	42
*L. interrogans*	Bataviae	UT0229	85	85	40	85	85	72
*L. interrogans*	Canicola	NR-20170*	70	74	20	64	70	42
*L. interrogans*	Medanensis	NR-20178*	78	80	13	80	72	64
*L. interrogans*	Pyrogenes	NR-20157*	80	44	10	80	80	44
*L. borgpetersenii*	Javanica	NR-20151*	80	80	20	76	76	62
*L. kirschneri*	Grippotyphosa	NR-20327*	76	76	19	76	82	64
*L. weilii*	Mengdeng	NR-20181*	85	85	18	85	85	72
Species	Chloramphenicol†	Ciprofloxacin†	Doxycycline†	Gentamicin†	Nitrofurantoin†	Piperacillin/tazobactam†	Tetracycline†	Doripenem‡
*Leptospira interrogans*	49	76	68	34	74	85	73	85
*L. interrogans*	64	85	66	33	85	85	74	85
*L. interrogans*	44	42	22	20	40	69	43	73
*L. interrogans*	75	74	64	32	38	72	66	85
*L. interrogans*	32	52	34	25	28	50	32	60
*L. interrogans*	50	62	24	30	34	74	38	78
*L. interrogans*	28	36	38	25	42	80	42	30
*L. borgpetersenii*	30	67	38	30	32	80	28	86
*L. kirschneri*	42	38	35	37	76	80	55	80
*L. weilii*	42	52	50	26	62	70	61	70
Species	Azithromycin§	Clindamycin§	Linezolid§	Penicillin§	Fosfomycin†	Nalidixic acid†	Trimethoprim sulfamethoxazole†	Rifampicin§
*Leptospira interrogans*	85	64	75	76	0	0	0	0
*L. interrogans*	85	60	85	67	0	0	0	0
*L. interrogans*	72	24	50	50	0	0	0	0
*L. interrogans*	85	51	72	65	0	0	0	0
*L. interrogans*	62	26	26	40	0	0	0	0
*L. interrogans*	76	35	78	70	0	0	0	0
*L. interrogans*	70	40	72	54	0	0	0	0
*L. borgpetersenii*	76	30	40	76	0	0	0	0
*L. kirschneri*	70	34	74	59	0	0	0	0
*L. weilii*	85	50	60	61	0	0	0	0

* NR represents strains deposited with Biodefense and Emerging Infections Research Resources Repository (*N* = 7).

Clinical and Laboratory Standards Institute (CLSI) threshold zone sizes for † Enterobacteriaceae, ‡ *Pseudomonas aeruginosa*, and § *Staphylococcus* spp.
