# Supplementary material for: Antimicrobial Disk Susceptibility Testing of Leptospira spp. Using Leptospira Vanaporn Wuthiekanun (LVW) Agar
Source: Am J Trop Med Hyg. 2015 Aug 5;93(2):241–3. doi: 10.4269/ajtmh.15-0180 (PMC4530741; doi:10.4269/ajtmh.15-0180)
Supplement: Supplementary file 1 [file SD2.pdf]

Supplemental Table 1

Reference zone diameter breakpoints of CLSI

Antimicrobials	Breakpoint for each antibiotic		
	S	I	R
Amoxicillin/clavulanic acid	≥ 18	14–17	≤ 13
Amoxicillin	≥ 17	14–16	≤ 13
Aztreonam	≥ 21	18–20	≤ 17
Cefoxitin	≥ 18	15–17	≤ 14
Ceftazidime	≥ 21	18–20	≤ 17
Ceftriaxone	≥ 23	20–22	≤ 19
Chloramphenicol	≥ 18	13–17	≤ 12
Ciprofloxacin	≥ 21	16–20	≤ 15
Doxycycline	≥ 14	11–13	≤ 10
Gentamicin	≥ 15	13–14	≤ 12
Nitrofurantoin	≥ 17	15–16	≤ 14
Piperacillin/tazobactam	≥ 21	18–20	≤ 17
Tetracycline	≥ 15	12–14	≤ 11
Doripenem	≥ 19	16–18	≤ 15
Azithromycin	≥ 18	14–17	≤ 13
Clindamycin	≥ 21	15–20	≤ 14
Linezolid	≥ 21	–	≤ 20
Penicillin	≥ 29	–	≤ 28
Fosfomycin	≥ 16	13–15	≤ 12
Nalidixic acid	≥ 19	14–18	≤ 13
Trimethoprim/sulfamethoxazole	≥ 16	11–15	≤ 10
Rifampicin	≥ 20	17–19	≤ 16

CLSI = Clinical and Laboratory Standards Institute.
